# Supplementary material for: A qualitative exploration of the educational needs of people living with heart failure: BANDAIDD-Explore study
Source: PLoS One. 2025 Feb 7;20(2):e0314059. doi: 10.1371/journal.pone.0314059 (PMC11805352; doi:10.1371/journal.pone.0314059)
Supplement: S1 Appendix — (DOCX) [file pone.0314059.s001.docx]

**Appendix 1: BANDAIDD Study Team Investigators**

Medical Research Future Fund – 2020 Cardiovascular Health Mission Grant (Ref: APP 2009251)

Digital solutions for heart failure best practice care

**Chief Investigators**

Professor Anthony Keech, University of Sydney & Royal Prince Alfred Hospital, Sydney

Dr Sean Lal, University of Sydney & Royal Prince Alfred Hospital, Sydney

Professor Peter Macdonald, University of New South Wales & St Vincent’s Hospital, Sydney

Professor Caleb Ferguson, University of Wollongong & Blacktown Hospital, Sydney

Mr Christopher Ryan, University of Melbourne

Professor Alicia Jenkins, University of Sydney

Dr Kathleen Dempsey, University of Sydney

Professor Clara Chow, University of Sydney & Westmead Allied Research Centre, Westmead Hospital, Sydney

Dr Rachel O’Connell, University of Sydney

Associate Professor Gary Kilov, University of Melbourne

**Associate Investigators**

Ms Rebecca Mister, NHMRC Clinical Trials Centre, University of Sydney

Professor Sandy Middleton, Australian Catholic University & St Vincent’s Hospital, Sydney

Dr Douglas Drak, University of Sydney

Professor Jo-Dee Lattimore, University of Sydney & Royal Prince Alfred Hospital, Sydney

Dr Andrzej Januszewski, University of Sydney

**Consumers**

Ms L-J L

Mr G B
