# Supplementary material for: A qualitative exploration of the educational needs of people living with heart failure: BANDAIDD-Explore study
Source: PLoS One. 2025 Feb 7;20(2):e0314059. doi: 10.1371/journal.pone.0314059 (PMC11805352; doi:10.1371/journal.pone.0314059)
Supplement: Attachment — Submitted filename: Response to Reviewers.docx [file pone.0314059.s003.docx]

| Reviewer | Comment | Action or response |
| --- | --- | --- |
| Editor | 1. Please ensure that your manuscript meets PLOS ONE's style requirements, including those for file naming. The PLOS ONE style templates can be found at  https://journals.plos.org/plosone/s/file?id=wjVg/PLOSOne_formatting_sample_main_body.pdf and  https://journals.plos.org/plosone/s/file?id=ba62/PLOSOne_formatting_sample_title_authors_affiliations.pdf | We have guided the structure of the document with these resources. Thank you |
|  | 2. Thank you for stating the following financial disclosure:  "Prof Caleb Ferguson is supported by a National Health and Medical Research Council 2020 Investigator (Emerging Leadership) Grant 2020–2025 Ref: 1196262. Australian government’s Medical Research Future Fund. Prof Anthony Keech is supported by National Health and Medical Research Council Senior Principal Research Fellowships (APP 1137071, & APP 2018537) and an NHMRC Program Grant (APP 1105467). Prof Clara Chow is supported by a National Health and Medical Research Leadership Investigator Grant (APP 11195326). This study was funded by the Medical Research Future Fund (MRFF) Cardiovascular Mission Grant Ref: 2009251; Digital solutions for heart failure best practice care."    Please state what role the funders took in the study. If the funders had no role, please state: ""The funders had no role in study design, data collection and analysis, decision to publish, or preparation of the manuscript.""  If this statement is not correct you must amend it as needed.  Please include this amended Role of Funder statement in your cover letter; we will change the online submission form on your behalf. | This has been amended as requested. |
|  | 3. We note that your Data Availability Statement is currently as follows: All relevant data are within the manuscript and its Supporting Information files.    Please confirm at this time whether or not your submission contains all raw data required to replicate the results of your study. Authors must share the “minimal data set” for their submission. PLOS defines the minimal data set to consist of the data required to replicate all study findings reported in the article, as well as related metadata and methods (https://journals.plos.org/plosone/s/data-availability#loc-minimal-data-set-definition).    For example, authors should submit the following data:    - The values behind the means, standard deviations and other measures reported;  - The values used to build graphs;  - The points extracted from images for analysis.    Authors do not need to submit their entire data set if only a portion of the data was used in the reported study.    If your submission does not contain these data, please either upload them as Supporting Information files or deposit them to a stable, public repository and provide us with the relevant URLs, DOIs, or accession numbers. For a list of recommended repositories, please see https://journals.plos.org/plosone/s/recommended-repositories.    If there are ethical or legal restrictions on sharing a de-identified data set, please explain them in detail (e.g., data contain potentially sensitive information, data are owned by a third-party organization, etc.) and who has imposed them (e.g., an ethics committee). Please also provide contact information for a data access committee, ethics committee, or other institutional body to which data requests may be sent. If data are owned by a third party, please indicate how others may request data access. | Due to the sensitive nature of the data collected for this study, requests to access the data set from qualified researchers trained in human subject confidentiality protocols may be sent to the corresponding author. The data are not publicly available due to containing information that could compromise the privacy of research participants. |
|  | 4. One of the noted authors is a group or consortium BANDAIDD Study Investigator team. In addition to naming the author group, please list the individual authors and affiliations within this group in the acknowledgments section of your manuscript. Please also indicate clearly a lead author for this group along with a contact email address. | This change has been incorporated as recommended. |
|  | 5. Please include a copy of Table 1 & 2 which you refer to in your text on page 4. | These are included in the supplementary materials. |
|  | 6. Please include captions for your Supporting Information files at the end of your manuscript, and update any in-text citations to match accordingly. Please see our Supporting Information guidelines for more information: http://journals.plos.org/plosone/s/supporting-information. | This has been amended as prescribed. |
|  | 7. Please review your reference list to ensure that it is complete and correct. If you have cited papers that have been retracted, please include the rationale for doing so in the manuscript text, or remove these references and replace them with relevant current references. Any changes to the reference list should be mentioned in the rebuttal letter that accompanies your revised manuscript. If you need to cite a retracted article, indicate the article’s retracted status in the References list and also include a citation and full reference for the retraction notice. | The citation list is correct and up to date. |
| Reviewer 1 | Reviewer #1: The study from Ferguson and colleagues presents a qualitative exploration of the educational needs of individuals living with heart failure (HF), their caregivers, and cardiovascular clinicians in New South Wales, Australia. This study identifies key themes regarding educational gaps and proposes improvements for patient-centered education to enhance self-management and overall care. The study is well-written and interesting with some minor revisions that should be considered by the authors: | Thank you. |
|  | **Abstract** Line 88: "Priorities identified for heart failure education include: timing of education, tailoring education to support the diverse needs of patients and the importance of continuity of care." The colon after "include" should be checked for consistency with other lists in the manuscript. | Thank you, this has been updated to reflect the manuscript. |
|  | **Introduction** Line 94: "Heart failure (HF) is a common, progressive, and debilitating syndrome affecting approximately 64 million people worldwide (1)." Consider rephrasing to "Heart failure (HF) is a common, progressive, and debilitating syndrome, affecting approximately 64 million people worldwide (1)." | Thank you, we have paraphrased as recommended. |
|  | Line 96: "In Australia, 1.5-2% of the national health expenditure is contributed by HF with hospital admissions accounting for two-thirds of these costs (2)." This could be clearer as "In Australia, HF contributes 1.5-2% of the national health expenditure, with hospital admissions accounting for two-thirds of these costs (2)." | Thank you, this has been updated as recommended. |
|  | Include explicit research questions or hypotheses | These are listed at the end of the introduction. |
|  | **Methods** Line 134: "Purposive sampling was used to recruit cardiovascular clinicians, people living with HF and their caregivers." This might read better as "Purposive sampling was used to recruit cardiovascular clinicians, people living with HF, and their caregivers.” | This has been updated as recommended. |
|  | Line 138: "Community-dwelling adults living with HF and their caregivers were invited to participate through study posters placed in the HF outpatient clinics of participating hospitals." Consider rephrasing for clarity. | This statement has been altered to include an oxford comma. |
|  | Provide more detail on the coding process and consensus-building in thematic analysis. | The detail in the manuscript is adequate for a scholarly research article. |
|  | **Results** Line 178: "Seven key themes were identified: (i) Understanding and reinforcing the signs and symptoms, self-management, medications, and prognosis and severity of HF; (ii) Providing concise and timely education; (iii) Building trust and relationships (iv) Accessibility of education to support patient needs; (v) Engaging family members and informal caregivers; (vi) Tailoring education to patients diverse needs; and (vi) Navigating the health system and dealing with continuity of care." Correct the repeated "vi" and ensure all themes are consistently listed with punctuation. | Thank you, this has been fixed. |
|  | **Conclusion** Line 531: "The clinical implications of this work are to prioritise self-management." Consider rephrasing to "The clinical implications of this work emphasize the prioritization of self-management." | Thank you, updated as recommended. |
|  | Emphasize the broader implications of the findings for policy in the Australian context and elsewhere. | This has been incorporated in the discussion. |
|  | For the COREQ checklist many items are listed as NA, please check if it is possible to add this information in th text, otherwise state them as limits of the study. | This has been checked and updated as much as applicable. These are not necessarily limitations of the study, given that COREQ guidelines are produced to be used for qualitative research in all contexts and disciplines, not every point here is relevant to the current project. It would not necessarily be feasible or relevant to have included all information in the reporting criteria. |
|  | The study can benefit from a central figure highlighting the seven themes emerged in the analysis State clearly that tables 1 and 2 are in supplementary materials. | This has been rephrased to be made more clear. |
| Reviewer 2. | Reviewer #2: Great subject in particular knowing that patients in your country can get home visits. Interesting that you collected data from the healthcare providers, patient with HF and their caregiver, so this provides a better understanding of each one experience with this disease. Your manuscript is relevant for anyone delivering care to these patients. | Thank you. |
|  | it is recommended to add more details/emphasis about the mHealth as it should be added to the discussion/results; more information and evidence are available in particular with HF patients in the Canadian context. | Further discussion has been added.  This is further discussed in another paper, which has been accepted into JMIR Cardio. (<https://pubmed.ncbi.nlm.nih.gov/39268614/>) Which is stage two of this studies. |
|  | the methodology lacks information about the types of questions you did ask because you were targeting 2 different types of respondents/participants. in addition, you did not complete all the steps or rather explain ensuring the rigor of your analysis. | This has been rephrased so it is clearly understood the focus group guides are in the supplementary materials, tables 1 and 2. |
|  | The abstract does not have the purpose of the study as you start right away with the methodology; details about the purpose should be added. | This has now been included, thank you. |
